# The effect of diabetes mellitus on tuberculosis in eastern China: A decision‐tree analysis based on a real‐world study

**DOI:** 10.1111/1753-0407.13444

**Published:** 2023-07-11

**Authors:** Yuxiao Ling, Xinyi Chen, Meng Zhou, Mengdie Zhang, Dan Luo, Wei Wang, Bin Chen, Jianmin Jiang

**Affiliations:** ^1^ School of Public Health, Health Science Center Ningbo University Ningbo China; ^2^ Department of Tuberculosis Control and Prevention Zhejiang Provincial Center for Disease Control and Prevention Hangzhou China; ^3^ Zhejiang University School of Public Health Hangzhou China; ^4^ Department of Public Health Hangzhou Medical College Hangzhou China; ^5^ Key Laboratory of Vaccine Prevention and Control of Infectious Disease of Zhejiang Province Hangzhou China

**Keywords:** comorbidity, decision trees, diabetes mellitus, pulmonary, tuberculosis, 共病, 决策树, 糖尿病, 肺结核

## Abstract

**Objectives:**

The public health system faces major challenges due to the double burden of diabetes mellitus (DM) and tuberculosis (TB) in China. We aimed to investigate the prevalence and impact of diabetes on patients with TB.

**Methods:**

Stratified cluster sampling was used to select 13 counties as study sites in the Zhejiang province. Patients who visited designated TB hospitals in these areas participated in this study between 1 January 2017 and 28 February 2019. Multiple logistic regression models were performed to investigate the association between DM and bacteriological and imaging results. A decision tree was used to predict the bacteriology and imaging results under the influence of DM.

**Results:**

Of 5920 patients with newly diagnosed pulmonary tuberculosis, 643 (12.16%) had DM. Patients with pulmonary TB and DM were more likely to have pulmonary cavities (adjusted odds ratio [aOR], 2.81; 95% confidence intervals [95% CI]: 2.35–3.37) and higher rates of positive bacteriological tests (aOR, 2.32; 95% CI:1.87–2.87). Decision‐tree analysis showed similar results.

**Conclusions:**

Concurrence of DM and pulmonary TB makes patients more likely to have positive bacteriological results and pulmonary cavities. Therefore, appropriate measures are necessary to promptly identify and manage patients with TB and DM.

## Graphical abstract

**Highlights**

- Diabetes can increase the severity of pulmonary tuberculosis.
- Patients with pulmonary tuberculosis and diabetes were more likely to have pulmonary cavities.
- Patients with pulmonary tuberculosis and diabetes may have higher rates of positive bacteriological tests.

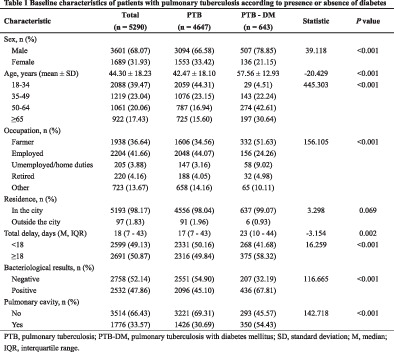

## 摘要

目的:由于糖尿病(DM)和肺结核(TB)在中国的双重负担, 公共卫生系统面临着重大挑战。我们旨在调查糖尿病患者中肺结核的患病率和影响。

方法:在浙江省选择了13个县作为研究地点, 采用分层集群抽样的方法。在这些地区指定的肺结核医院就诊的患者参与了这项研究, 研究时间为2017年1月1日至2019年2月28日。进行多项逻辑回归模型研究DM与细菌学和影像学结果之间的关联性。使用决策树预测在DM影响下的细菌学和影像学结果。

结果:在5920例新诊断的肺结核患者中, 有643例(12.16%)患有DM。同时患有肺结核和DM的患者更可能出现肺空洞(调整后的比值比[aOR]为2.81；95%置信区间[95% CI]为2.35‐3.37), 并且细菌学检测阳性率更高(aOR为2.32；95% CI为1.87‐2.87)。决策树分析显示出类似结果。

结论:同时患有肺结核和DM的患者更容易出现细菌学检测结果阳性和肺空洞。有必要采取适当措施及时识别和管理同时患有TB和DM的患者。

## 1 INTRODUCTION

Tuberculosis (TB) is a preventable and curable disease caused by *Mycobacterium tuberculosis* (*MTB*). However, it remains a major public health problem with increasing morbidity and mortality over the past 2 years.^1^ According to the Global Tuberculosis Report 2022 released by the World Health Organization, there were an estimated 10.6 million TB cases globally in 2021, with a 4.5% year‐on‐year increase. It is worth noting that two thirds of the total TB cases occur in eight countries, of which China ranks third (7.4%) and is still in the middle of an epidemic of TB. In China, an estimated 780 000 people have newly diagnosed TB, with an incidence rate of 55 per 100 000. Meanwhile, the estimated number of new and relapse cases was 585 340, of which pulmonary tuberculosis (PTB) accounted for 95%.^1^ TB cases in China were attributable to the following risk factors: smoking, alcohol use disorders, undernutrition, diabetes, and HIV.

Diabetes mellitus (DM) is a noncommunicable metabolic disorder that can be attributed to various factors. Globally, the burden of DM has been increasing at an alarming rate. China, one of the countries with the highest burden of DM worldwide, has an estimated 12.4% prevalence of DM among adults.^2^ According to the International Diabetes Federation, China is the country with the most people with diabetes, with an estimated number of adults with DM to exceed 140 million in 2021, double the number of the next country on the list, India. And more than 174 million people will have DM by 2045.^3^

An estimated 370 000 new TB cases were associated with DM globally in 2021.^1^ TB has high incidence and prevalence in patients with DM.^4^ Jeon and Murray reported that the risk of TB was three times higher in patients with DM than in those without DM.^5^ Meanwhile, a meta‐analysis reported a 15.3% prevalence of DM among 2.3 million patients with TB.^6^ These two diseases are closely related and promote one another. A high‐glucose environment and an impaired immune system^7^ accelerate the reproduction rate of *MTB* and promote the production of drug‐resistant strains.^8^ Furthermore, the occurrence of DM can aggravate the clinical manifestations of PTB and delay the conversion of sputum smears and cultures^9^ causing an even higher possibility of treatment failure and recurrence.^10^ It may even aggravate glucose metabolism disorders and increase the incidence of ketoacidosis.^11^ In addition, DM may also double the median cost of treatment of patients with TB.^12^, ^13^ The presence of comorbidities with TB and DM poses a serious threat to TB control.

Despite increasing evidence of the effect of DM on the risk and outcomes of TB, global data on patients with TB and DM are limited and variable.^14^, ^15^ Moreover, studies on TB‐DM in China are limited despite the high burden of TB‐DM. In this study, we investigated the prevalence of DM in patients with TB from Zhejiang Province. We studied the impact of DM on the bacteriological and imaging manifestations of PTB to assist in the prevention and early identification of PTB‐DM in China.

## 2 METHODS

### 2.1 Study setting

Zhejiang Province is located in the eastern coastal region of China and consists of 11 regional cities containing nearly 90 counties. According to the data of the seventh population census released by the Zhejiang Provincial Bureau of Statistics, there were over 64.5 million local residents and nearly 30 million migrant populations. Zhejiang has reported nearly three million TB cases in the past decade, with an incidence of 52.25/100 000 in 2018 alone.^16^ The age‐standardized incidence of DM was reported to be 281.73 per 100 000 person‐years during 2007–2017.^17^

### 2.2 Study design

Stratified cluster sampling was performed. Hangzhou and Wenzhou were selected as the study cities based on their location. Counties with relatively large annual outpatient visits from the two cities were selected as study sites based on the incidence of TB and socioeconomic conditions. Finally, 13 counties were selected as study sites. From 1 January 2017 to 28 February 2019, all patients with active TB diagnosed and reported by designated TB hospitals in the survey area were investigated, and the screening was completed. Further information was collected from patients with active PTB who met the screening criteria.

### 2.3 Inclusion and exclusion criteria

The inclusion criteria were as follows: (a) patients aged 18 years or older, (b) patients newly diagnosed with PTB, (c) patients who comply with treatment, and (d) patients receiving daily treatment regimens.

The exclusion criteria were as follows: the presence of (a) HIV infection, (b) pleurisy, (c) silicosis, (d) extrapulmonary TB, (e) tracheal and bronchial TB, (f) communication disorder, or (g) incarceration.

Participants were included when all inclusion criteria were met and none of the exclusion criteria were present.

### 2.4 Data collection

During the patient's visit, trained and qualified medical staff collected data from the Tuberculosis Information Management Systems of Zhejiang Province, and from the clinical diagnosis and treatment reports from eligible respondents. The information collected included (a) general characteristics (age, biological sex, residence, and occupation); (b) clinical information (imaging manifestations, eg, pulmonary cavities; bacteriological data, eg, sputum smear results; and comorbidities, eg, DM status); and (c) health services‐related data (time to onset of PTB symptoms, time to first health care visit, and time to PTB diagnosis). Total diagnostic delay was assessed by asking patients to recall the time from the onset of PTB symptom(s) to the first visit to a health facility and the time from the patient's first visit to a health facility to confirm the TB diagnosis. These data were collected and recorded by health workers at the time of TB registration. Similarly, DM information was routinely screened by PTB patient self‐reports during consultations.

### 2.5 Study definitions

Patients with PTB confirmed by laboratory and clinical diagnosis who met the National Diagnostic Criteria for Pulmonary Tuberculosis (WS 288–2017) and the Classification of Tuberculosis criteria (WS196–2017) were included in the study. Laboratory diagnosis was based on the result of detectable acid‐fast bacilli obtained from sputum smears and/or sputum culture or a positive result from the molecular diagnostic instrument (eg, GeneXpert). Clinical diagnosis was based on chest radiographs, epidemiological surveys, clinical symptoms, and other relevant tests.

According to the Chinese Guidelines for the Prevention and Treatment of Type 2 Diabetes (2013), patients with TB were considered to have DM if they had been diagnosed with DM or were taking drugs to control blood glucose at the time of the medical consultation.

In this study, the total diagnostic delay (hereinafter referred to as total delay) was defined as the time interval between the onset of PTB symptom(s) and the date of diagnosis. The median total delay was used to dichotomize the delay into “long total delay” (greater than or equal to the median) or “acceptable total delay” (less than the median).

### 2.6 Statistical analysis

SPSS 23.0 (IBM Corporation, Armonk, NY, USA) and R 4.2.2 were used to perform the analyses. Continuous variables are reported as mean ± SD or median and interquartile range (IQR). Categorical variables are reported as the number of cases (n) and percentage (%). We compared baseline data between patients with and without DM (PTB‐DM and PTB, respectively) using the chi‐square test for categorical variables and Mann‐Whitney *U* test for continuous variables. Univariate analysis of bacteriological results and pulmonary cavities was performed for all patients with PTB. Multivariate logistic regression analysis was performed, accounting for DM status for all patients with PTB and adjusting for sex, age, occupation, and total delay. On this basis, hierarchical logistic regression analysis corresponding to different adjustment factors was performed, and the results are presented as forest plots. Odds ratios (OR) and 95% confidence intervals (95% CI) were calculated using logistic regression analyses with different adjustment levels. *p* values were two tailed with statistical significance set at .05.

A classification decision‐tree model based on the chi‐square automatic interaction detector (CHAID) was established with the bacteriological and imaging results as the dependent variable, and DM (forced as the first variable), sex, age, residence, occupation, and total delay as the independent variables. The CHAID tree‐growing method selects the independent variable with the strongest interaction with the dependent variables. Chi‐square or likelihood ratio chi‐square tests were used to determine the best category merging and splitting points of the decision tree. The significance level for the splitting point was a *p* value of <.05. The maximum tree depth was set at three. The minimum number of cases was set to 200 for parent nodes and 100 for child nodes. Simultaneously, we randomly divided the participants into two groups: One group was used as the training set to build the decision‐tree model (consisting of 80% of the total patients), and the other group was used to verify the accuracy of the model (the remaining 20%).

## 3 RESULTS

### 3.1 Baseline characteristics of study participants

**FIGURE 1:**
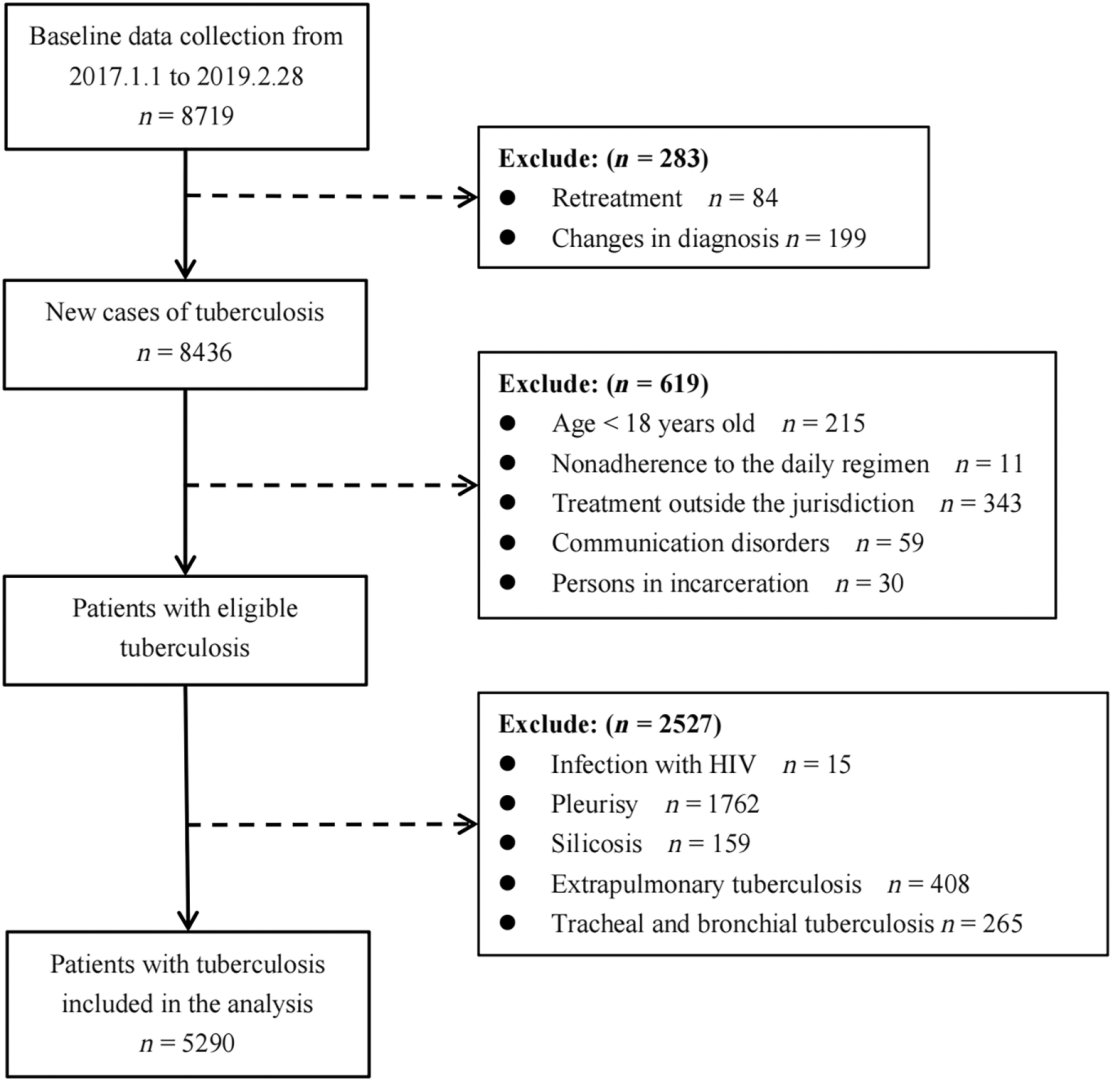
Flow diagram of participant selection.

In this study, 8719 patients visited the designated TB hospitals and participated in the survey between 1 January 2017 and 28 February 2019. Overall, 5920 patients with PTB were enrolled in the study after excluding ineligible patients (Figure 1). The mean age of patients with PTB was 44.30 ± 18.23 years where 17.43% were >65 years old, 68.07% were males, and 98.17% were local residents. The median total delay of study participants was 18 days (IQR: 7–43). Positive bacteriological results were observed in 47.86% of the patients, and 33.57% had pulmonary cavities (Table 1).

**TABLE 1:** Baseline characteristics of patients with pulmonary tuberculosis according to presence or absence of diabetes.

Characteristic	Total	PTB	PTB‐DM	Statistic	*p* value
	(*n* = 5290)	(*n* = 4647)	(*n* = 643)		
Sex, *n* (%)					
Male	3601 (68.07)	3094 (66.58)	507 (78.85)	39.118	<.001
Female	1689 (31.93)	1553 (33.42)	136 (21.15)		
Age, years (mean ± SD)	44.30 ± 18.23	42.47 ± 18.10	57.56 ± 12.93	−20.429	<.001
18–34	2088 (39.47)	2059 (44.31)	29 (4.51)	445.303	<.001
35–49	1219 (23.04)	1076 (23.15)	143 (22.24)		
50–64	1061 (20.06)	787 (16.94)	274 (42.61)		
≥65	922 (17.43)	725 (15.60)	197 (30.64)		
Occupation, *n* (%)					
Farmer	1938 (36.64)	1606 (34.56)	332 (51.63)	156.105	<.001
Employed	2204 (41.66)	2048 (44.07)	156 (24.26)		
Umemployed/home duties	205 (3.88)	147 (3.16)	58 (9.02)		
Retired	220 (4.16)	188 (4.05)	32 (4.98)		
Other	723 (13.67)	658 (14.16)	65 (10.11)		
Residence, *n* (%)					
In the city	5193 (98.17)	4556 (98.04)	637 (99.07)	3.298	.069
Outside the city	97 (1.83)	91 (1.96)	6 (0.93)		
Total delay, days (M, IQR)	18 (7–43)	17 (7–43)	23 (10–44)	−3.154	.002
<18	2599 (49.13)	2331 (50.16)	268 (41.68)	16.259	<.001
≥18	2691 (50.87)	2316 (49.84)	375 (58.32)		
Bacteriological results, *n* (%)					
Negative	2758 (52.14)	2551 (54.90)	207 (32.19)	116.665	<.001
Positive	2532 (47.86)	2096 (45.10)	436 (67.81)		
Pulmonary cavity, *n* (%)					
No	3514 (66.43)	3221 (69.31)	293 (45.57)	142.718	<.001
Yes	1776 (33.57)	1426 (30.69)	350 (54.43)		

Abbreviations: IQR, interquartile range; M, median; PTB, pulmonary tuberculosis; PTB‐DM, pulmonary tuberculosis with diabetes mellitus.

### 3.2 Baseline characteristics of patients with PTB with and without DM

The prevalence of PTB‐DM was 12.16% among patients newly diagnosed with TB. As described in Table 1, the patients with PTB‐DM were mainly males (78.85% vs. 66.58%, *p* < .001), older aged (57.56 ± 12.93, *p* < .001), predominantly farmers (51.63%, *p* = .002), with longer median total delay (23 vs. 17, *p* < .001), and a greater incidence of positive bacteriological results (67.81% vs. 45.10%, *p* < .001), and pulmonary cavities (54.43% vs. 30.69%, *p* < .001), when compared with patients who have PTB without DM.

### 3.3 Univariate analysis of bacteriological results and imaging results in patients with PTB

Data on patients with PTB was included in the univariate analysis (Table 2). Regarding bacteriological results, there were significant differences in sex, age, occupation, and total delay. The association between PTB and residence was not statistically significant. There were statistically significant differences in sex, occupation, and total delay among patients with pulmonary cavities and those without.

**TABLE 2:** Comparison of bacteriological results and imaging results in patients with pulmonary tuberculosis.

Characteristic	Bacteriological results				Pulmonary cavity			
	Negative	Positive	Statistic	*p* value	No	Yes	Statistic	*p* value
Sex, *n* (%)								
Male	1830 (66.35)	1771 (69.94)	7.838	.005	2253 (64.11)	1348 (75.90)	75.40	<.005
Female	928 (33.65)	761 (30.06)			1261 (35.89)	428 (24.10)		
Age, *n* (%)								
18–34	1241 (45.00)	847 (33.45)	163.466	<.001	1403 (39.93)	685 (38.57)	5.856	.119
35–49	673 (24.40)	546 (21.56)			811 (23.08)	408 (22.97)		
50–64	523 (18.96)	538 (21.25)			673 (19.15)	388 (21.85)		
≥65	321 (11.64)	601 (23.74)			627 (17.84)	295 (16.61)		
Occupation, *n* (%)								
Farmer		1069 (42.22)	99.710	<.001	1191 (33.89)	747 (42.06)	45.263	<.001
Employed	1313 (47.61)	891 (35.19)			1544 (43.94)	660 (37.16)		
Umemployed/home duties	85 (3.08)	120 (4.74)			158 (4.50)	47 (2.65)		
Retired	112 (4.06)	108 (4.27)			142 (4.04)	78 (4.39)		
Other	379 (13.74)	344 (13.58)			479 (13.63)	244 (13.74)		
Residence, *n* (%)								
In the city	2711 (98.30)	2482 (98.03)	0.537	.464	3452 (98.24)	1741 (98.03)	0.279	.597
Outside the city	47 (1.70)	50 (1.97)			62 (1.76)	35 (1.97)		
Total delay, *n* (%)								
<18	1589 (57.61)	1010 (39.89)	165.942	<.001	1785 (50.80)	814 (45.83)	11.629	.001
≥18	1169 (42.39)	1522 (60.11)			1729 (49.20)	962 (54.17)		

### 3.4 Association between DM and bacteriological and imaging results in patients with PTB

**FIGURE 2:**
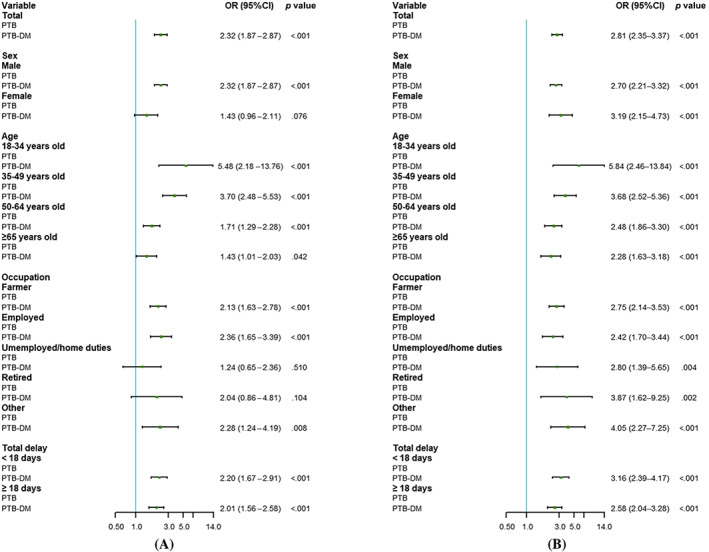
Association of diabetes mellitus with bacteriological results and imaging results in patients with pulmonary tuberculosis. (A) Bacteriological results. (B) Imaging results. Sex, age, occupation, and total delay were adjusted in the total population model. The adjustment factors of each hierarchical model deleted the corresponding classification factors from the four factors. DM, diabetes mellitus; OR, odds ratio; PTB, pulmonary tuberculosis.

When age, sex, occupation, and total delay were considered, the model showed that the association of PTB‐DM with positive bacteriological results (OR = 2.32, 95% CI: 1.87–2.87) was statistically significant (Figure 2A). The presence of DM was associated with positive bacteriological results when patients were stratified by sex, age, occupation, and total delay while maintaining other adjustment factors as follows: men (OR = 2.32, 95% CI: 1.87–2.87), different age groups (18–34 years old, OR = 5.48, 95% CI: 2.18–13.76; 35–49 years old, OR = 3.70, 95% CI: 2.48–5.53; 50–64 years old, OR = 1.71, 95% CI: 1.29–2.28; ≥ 65 years old, OR = 1.43, 95% CI: 1.01–2.03), farmers (OR = 2.13, 95% CI: 1.63–2.78), active employees (OR = 2.36, 95% CI: 1.65–3.39), other occupations (OR = 2.28, 95% CI: 1.24–4.19), and in delay (<18 days, OR = 2.20, 95% CI: 1.67–2.91; ≥18 days, OR = 2.01, 95% CI: 1.56–2.58).

Similarly, in the total sample, the association between DM and pulmonary cavities was statistically significant (OR = 2.81, 95% CI: 2.35–3.37) in patients with PTB‐DM (Figure 2B). After controlling for different factors, there was also a statistically significant association between DM and pulmonary cavities in different sexes, age groups, occupations, and delay.

### 3.5 Decision‐tree model

The Tables S1 and S2 summarized the prediction results of the decision‐tree models. The sensitivity, specificity, and overall accuracy of the bacteriological results' decision‐tree model was 71.6.0%, 50.3%, and 60.0%, respectively. The decision tree of pulmonary cavities was 16.9%, 93.7%, and 68.1%, respectively.

**FIGURE 3:**
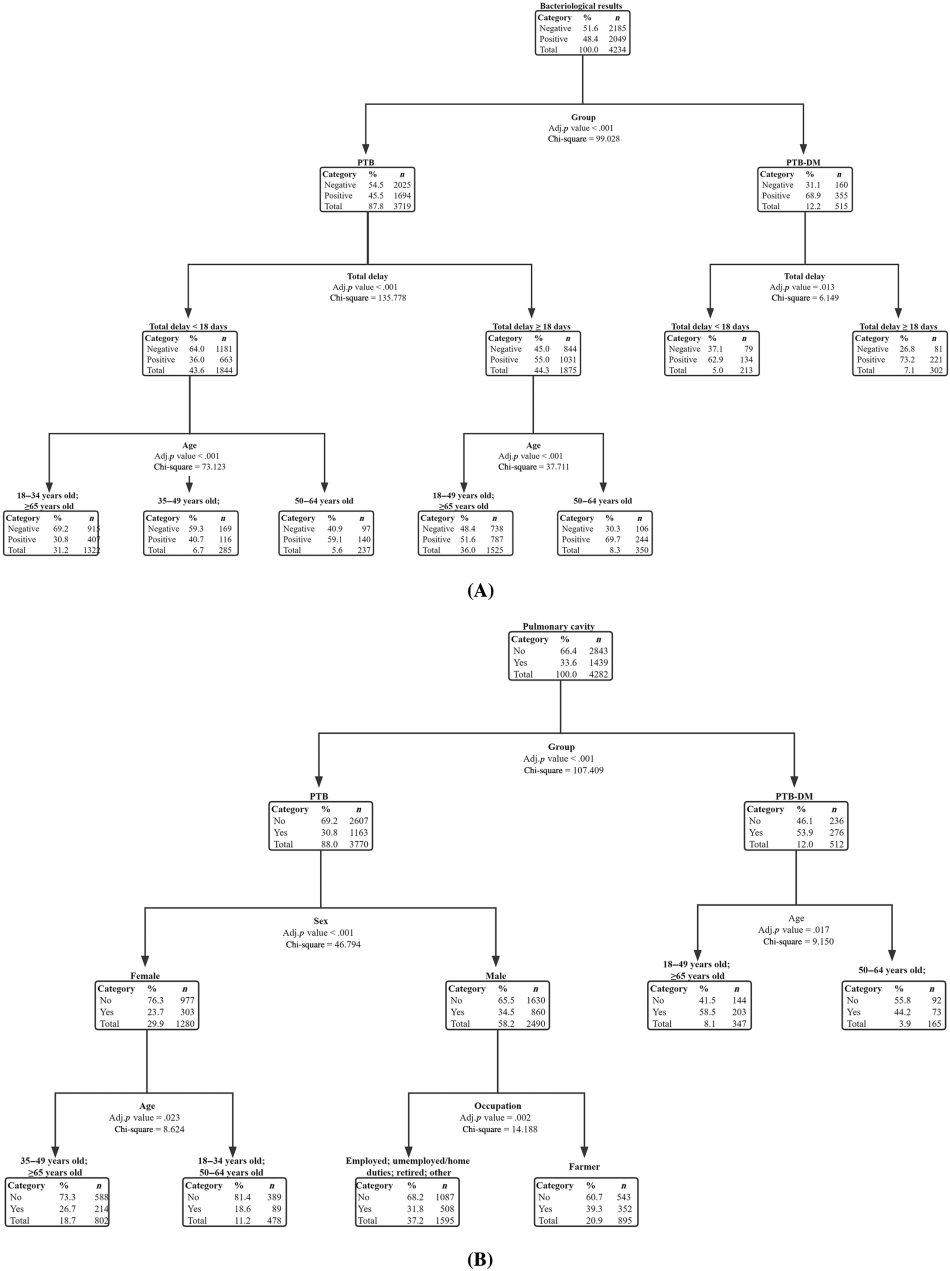
Classification decision trees of bacteriological results and imaging results for patients with pulmonary tuberculosis. (A) bacteriological results. (B) imaging results. DM, diabetes mellitus; PTB, pulmonary tuberculosis.

The decision‐tree model for positive risk assessment of bacteriological results is illustrated in Figure 3A. Among the five variables, DM was the most significant determining factor, which was located at the root of the decision tree and was presented as the first‐level split of two initial branches. Total delay and age were located in the second‐ and third‐level splits, respectively. When combined with DM, the total delay time is ≥18 days; In the absence of DM, a total delay time of ≥18 days, and the patient age between 50 and 64 years significantly increased the probability of positive bacteriological results in patients with PTB.

The decision‐tree model for positive risk assessment of pulmonary cavities is shown in Figure 3B. In patients with DM, and the age of patients between 18–49 and ≥65 years, significantly increased the probability of pulmonary cavities in PTB patients. However, in patients without DM, male sex and a farming occupation can significantly increase the probability of pulmonary cavities in PTB patients.

## 4 DISCUSSION

In the present study, we observed that the prevalence of DM was 12.16% in 5920 patients with newly diagnosed PTB. This implied that DM was relatively prevalent in patients with PTB in Zhejiang province. Moreover, the results illustrated that DM increased the risk of positive bacteriological results and pulmonary cavities in patients with PTB. Furthermore, the decision trees also suggested that DM might aggravate PTB severity.

The overall prevalence of DM among TB patients has been reported to be 7.8% in China, where the highest prevalence was observed in Northeast China (21.9%) among four economic regions, followed by East Coast (8.3%), Western China (5.9%), and Central China (5.1%).^18^ Inconsistencies in prevalence may be caused by regional economic differences and inequity in regional health systems. In Zhejiang province, the prevalence of DM observed in patients with in this study was much higher than that in the studies by Xiao et al (7.0%)^19^ and Wu et al (8.12%).^8^ This suggests that we should be aware that the prevalence of DM in patients with TB is gradually increasing. In addition, compared with the overall prevalence of DM in European patients with TB (7.5%),^6^ the prevalence of DM among patients with TB in China is higher. In contrast, its prevalence is lower than that in India (28%),^20^ Qatar (17%),^21^ and Saudi Arabia (27.1%).^22^ This may be attributed to the effort of the Chinese National Basic Public Health Service Program, but monitoring management and public awareness still require further improvement. Strengthen the integration of monitoring and evaluation to achieve horizontal‐vertical integration.^12^ Additionally, the differences between developed and developing countries deserve further consideration.

Notably, the proportion of DM among male patients with PTB was significantly higher than that among female patients (78.85% vs. 21.15%), which is similar to the results of other research reports.^23^, ^24^ This phenomenon may be related to the fact that DM itself is more prevalent in men. Differences in energy balance, metabolic regulation, and lifestyle make men more likely to develop obesity, insulin resistance, and hyperglycemia.^25^

Diabetes, as one of the risk factors, may promote the transition from latent to active TB. Susceptibility to TB in DM is associated not only with hyperglycemia and insulin resistance but also with macrophage and lymphocyte function.^26^ It is also strongly associated with impaired immune responses in patients with DM. Studies have found that the function of neutrophils, macrophages, natural killer cells, and some other components of innate immunity is seriously affected by metabolic alterations in DM.^27^ On the other hand, adaptive immunity may be delayed by damage to antigen‐presenting cell. T cell subsets are altered by dysregulated cytokine profile. B cell activation and antibody production are impaired.^7^ These changes promote bacterial escape, which is more likely to result in active TB.^28^ This suggests that patients with DM may be more susceptible to infection PTB.

PTB manifestations were more serious in patients with PTB and DM, which is consistent with previous studies.^12^ Patients with TB‐DM have been reported to have a higher prevalence of sputum smear positivity.^29^ In addition, patients with DM are more likely to have pulmonary cavities.^10^, ^21^, ^22^ Study results have shown that abnormal opacities over the lower lung field, pulmonary cavities, and cavity nodules were more frequent among patients with TB‐DM.^9^ Armstrong et al found that the probability of cavitary TB was as high as 70% and the probability of smear‐positive TB was as high as 50% in patients with DM according to the data shown in the 2010–2017 report of the American Tuberculosis Surveillance System.^11^ *MTB* induces robust cell‐mediated immune responses, resulting in the formation of pulmonary granulomas (tubercles), which prevent the spread of the pathogen.^30^ However, in people with DM have impaired immune systems, *MTB* is reactivated in the host and continues to replicate.^28^ Elevated levels of anti‐inflammatory cytokines and interleukin (IL)‐4, which inhibits the expression of interferon, may cause patients with DM to fail to control the growth of bacteria and have relatively high bacterial load before generating adaptive immune response.^31^ These granulomatous structures undergo central caseation and eventually rupture, causing the pathogen to spread to the airways. Therefore, this “TB cavity” is often associated with sputum smear positivity.^32^ In addition, the higher levels of IL‐7 and IL‐8 in patients with DM may lead to more granulocyte infiltration, and these byproducts of hyperglycemia combined with oxidative stress can induce a proinflammatory response, which can lead to more severe inflammation and TB.^33^ For example, patients with DM are more likely to develop drug resistance, prolong clinical treatment, and increase the likelihood of failure and recurrence.^34^, ^35^

We were able to control for the influence of different factors and found that patients with DM had more positive bacteriological results and pulmonary cavities than patients without DM. Previous studies have illustrated that sex, age, accessibility to health care (leading to a delay in patient presentation), and comorbidity could influence the presentation of TB.^36^ Our age‐stratified results showed that the ORs of positive bacteriological results and pulmonary cavities gradually decreased with increasing age. One study illustrated that the association between TB and DM is more prominent in younger people.^15^ Jeon and Murray found that the risk ratios (RR) for the age group >40 years decreased with increasing age, using age‐stratified RR.^5^ A similar result was reported by Makuka et al's study.^37^ This may be related to the increased participation of young people in social activities, which makes them more susceptible to TB infection and clinical manifestations. Meanwhile, in our stratified results, the ORs of bacteriological and imaging results were decreased in patients with a long total delay compared with patients with an acceptable total delay. Xiao et al reported that patients with TB and DM had a shorter patient delay and total delay.^19^ This may be related to the higher health awareness and regular treatment behaviors among patients with DM. In addition, a study in India found that the presence of DM is associated with a higher incidence of smear‐positive TB in urban areas than in rural areas.^38^

As a feature point of our decision‐tree model, DM was placed at the top of the tree. Although the specificity was low in the bacteriological decision‐tree model, it showed good sensitivity and could be used as a reference tool for TB screening. The imaging decision‐tree model showed low sensitivity and high specificity. Thus, this prediction model should be improved in future studies. Furthermore, our decision‐tree model consists of common and easily available predictors, making it an effective tool for disease prevention in health care.

The results of this study have several advantages. This study excluded the impact of other possible comorbidities such as HIV infection, pleurisy, silicosis, extrapulmonary TB, and tracheal and bronchial TB. Analyzing the impact of only DM in newly diagnosed patients with PTB made the results more referenced and targeted. However, this study has some limitations. First, we were not able to explore more complex relationships because of the shortage of some important variables, such as the glycemic index, smoking, and drinking. Second, the prevalence of DM was based on self‐reporting of the diagnosis by patients. Third, the types of diabetes in our study were not clear, which may limit the ability to explore the association between different types of diabetes and tuberculosis. Studies have found that type 1 DM has an acute onset and progresses rapidly, but the treatment effect is better than that of type 2 DM.^39^, ^40^ Further research is needed. Moreover, blood glucose tests were not performed in this study, which may have led to missed cases of DM and thus underestimated the prevalence of DM in patients with PTB. Finally, the conclusions drawn in this study may not be generalizable to other regions due to regional socioeconomic differences and access to health services. Therefore, more research is needed to confirm our results.

## 5 CONCLUSIONS

Our results show that the prevalence of diabetes is relatively common among patients with newly diagnosed PTB in Zhejiang Province. Diabetes may aggravate the bacteriological and imaging manifestations of PTB, which are more likely to lead to positive bacteriological results and pulmonary cavities. This suggests that corresponding measures should be taken to improve the early identification of concurrent diabetes and PTB, especially in areas with a double burden of both diseases. Early identification of high‐risk patients may reduce disease severity.

## CONFLICT OF INTEREST STATEMENT

The authors declare that the research was conducted in the absence of any commercial or financial relationships that could be construed as a potential conflict of interest.

## Supporting information


**Data S1.** Supporting Information.
