# Supplementary material for: The effect of diabetes mellitus on tuberculosis in eastern China: A decision‐tree analysis based on a real‐world study
Source: J Diabetes. 2023 Jul 11;15(11):920–30. doi: 10.1111/1753-0407.13444 (PMC10667642; doi:10.1111/1753-0407.13444)
Supplement: Supplementary file 1 — Data S1. Supporting Information. [file JDB-15-920-s001.docx]

**Supplementary Tables**

| **Table-e1 Prediction results of bacteriological results** | | | | | |
| --- | --- | --- | --- | --- | --- |
| Outcomes | Actual class | Observed | Predicted class | | Accuracy (%) |
|  |  |  | Negative | Positive |  |
| Bacteriological results | Training | Negative | 1084 | 1101 | 49.6% |
|  |  | Positive | 523 | 1526 | 74.5% |
|  |  | Overall |  |  | 61.6% |
|  | Test | Negative | 288 | 285 | 50.3% |
|  |  | Positive | 137 | 346 | 71.6% |
|  |  | Overall |  |  | 60.0% |

| **Table-e2 Prediction results of and pulmonary cavity** | | | | | |
| --- | --- | --- | --- | --- | --- |
| Outcomes | Actual class | Observed | Predicted class | | Accuracy (%) |
|  |  |  | No | Yes |  |
| Pulmonary cavity | Training | No | 2699 | 144 | 94.9% |
|  |  | Yes | 1236 | 203 | 14.1% |
|  |  | Overall |  |  | 67.8% |
|  | Test | No | 629 | 42 | 93.7% |
|  |  | Yes | 280 | 57 | 16.9% |
|  |  | Overall |  |  | 68.1% |
